# Deaths from Cysticercosis, United States

**DOI:** 10.3201/eid1302.060527

**Published:** 2007-02

**Authors:** Frank J. Sorvillo, Christopher DeGiorgio, Stephen H. Waterman

**Affiliations:** * University of California Los Angeles, Los Angeles, California, USA; † Department of Public Health, Los Angeles County, California, USA; ‡ University of California San Diego, San Diego, California, USA

**Keywords:** cysticercosis, mortality, epidemiology, surveillance, public health, research

## Abstract

Cysticercosis has emerged as a cause of severe neurologic disease in the United States. We evaluated cysticercosis-related deaths in the United States for 1990–2002 by race, sex, age, state of residence, country of birth, and year of death. A total of 221 cysticercosis deaths were identified. Mortality rates were highest for Latinos (adjusted rate ratio [ARR] 94.5, relative to whites) and men (ARR = 1.8). The mean age at death was 40.5 years (range 2–88). Most patients (187 [84.6%]) were foreign born, and 137 (62%) had emigrated from Mexico. The 33 US-born persons who died of cysticercosis represented 15% of all cysticercosis-related deaths. The cysticercosis mortality rate was highest in California, which accounted for ≈60% of all deaths. Although uncommon, cysticercosis is a cause of premature death in the United States. Fatal cysticercosis affected mainly immigrants from Mexico and other Latin American countries; however, US-born persons were also affected.

## Abstract 2

Most deaths occur among Latino immigrants; US-born persons are affected to a lesser extent.

Cysticercosis, a parasitic infection caused by the larval form of the pork tapeworm, *Taenia solium*, has been increasingly recognized as a cause of severe but preventable neurologic disease in the United States (*1*–*5*). Reports documenting hundreds of cases, mainly of neurocysticercosis, have drawn attention to this previously underrecognized disease (*6*,*7*).

Cysticercosis has a complex life cycle. The larval infection, cysticercosis, is transmitted through the fecal-oral route. Eggs from the adult tapeworm *T. solium*, which are directly infectious, are shed in the feces of a human tapeworm carrier and subsequently ingested by pigs, the usual intermediate host (*8*). The oncosphere embryos emerge from the eggs, penetrate the intestinal wall, and are disseminated by the bloodstream to various tissues where the larval stage, or cysticercus, develops. The cycle is completed when humans, the only naturally infected definitive host, consume raw or undercooked pork containing cysticerci, which attach to the small bowel and develop into the adult tapeworm. However, humans may also become infected with the larval stage when eggs are ingested, typically in contaminated food or water. Neurocysticercosis, the most severe form of the disease, occurs when larvae invade tissue of the central nervous system.

Cysticercosis in the United States affects mainly immigrants from Latin America, where the disease is endemic. However, cysticercosis acquired in the United States has been repeatedly documented over the past 15 years, and travel-related infection in US-born persons has been reported (*9*–*11*). Given the ongoing sizeable immigration from disease-endemic areas, cysticercosis will grow in clinical and public health importance; however, data on cysticercosis in the United States are lacking. The disease is not nationally reportable, few local jurisdictions require reporting, and surveillance systems for cysticercosis have rarely been implemented (*10*,*12*). In the absence of effective surveillance, the true prevalence of cysticercosis in the United States is largely unknown. Although several hospital-based series have provided valuable insights into the occurrence of cysticercosis, they reflect only a portion of actual cases and do not measure the true effect of the disease on the general population and at-risk populations. Moreover, few data exist on cysticercosis as a cause of death in the United States (*6*,*13*). To augment current information on the effect of cysticercosis in the United States, we evaluated national mortality records for cysticercosis-related deaths for the 13-year period 1990–2002.

## Methods

### Data Source

Mortality data were obtained from the National Center for Health Statistics (NCHS). Death certificates, which are required by state law, must indicate a cause or sequence of events that led to death, as determined by the attending physician. If a physician is not in attendance or the death is accidental or occurs under suspicious circumstances, then cause of death is determined by the local coroner or medical examiner. Death certificate data are transmitted from state jurisdictions to NCHS. The US Multiple Cause of Death Files for 1990 through 2002 were searched for listings of cysticercosis (ICD-9 code 123.1 for 1989–1998 and ICD-10 code B69 for 1999–2002). Availability of this national data source typically has a 3-year lag time. The multiple cause of death data contain all causes of death provided by the physician or coroner. Such information is more complete than data files with primary cause of death only. Additional variables extracted from the death record included age, sex, race/ethnicity, level of education, country of birth, place of death, date of death, and other concurrent conditions.

### Data Analysis

Cysticercosis mortality rates per million population were calculated. Population data were obtained from the US Census Bureau. Crude cysticercosis mortality rates and 95% confidence intervals (CIs) were computed by age group (<1, 1–4, 5–14, 15–24, 25–34, 35–44, 45–54, 55–64, 65–74, 75–84, ≥85 years), sex, race/ethnicity (white, black, Latino, Asian, Native American), and state of residence. Age-adjusted rates were calculated for race/ethnicity, sex, and state. The US population for the year 2000 was used as the standard population for all age-adjusted rates. Rate ratios, adjusted rate ratios, and 95% CIs were also computed. Demographic characteristics of US-born patients were compared with those of foreign-born patients. The χ^2^, Fisher exact, and Student *t* tests were used where appropriate to assess apparent differences. Conditions occurring with cysticercosis were examined and compared with a random sample of deaths from causes other than cysticercosis matched by patient age, sex, and race/ethnicity. Matched odds ratios and 95% CIs were calculated for each condition.

## Results

Over the 13-year study period, 221 cysticercosis deaths were identified, representing an annual age-adjusted mortality rate of 0.06 per million population (95% CI, 0.05–0.07). Most persons who died from cysticercosis (187 [84.6%]) were Latino; 15 (6.8%) were white, 13 (5.9%) were black, 5 (2.3%) were Asian, and 1 (0.5%) was Native American (Table 1). By sex, 137 (62.0%) were male, and 84 (38.0%) were female. Mean age at death was 40.5 years (range 2–88 years). Most persons who died (187 [84.6%]) were foreign born, and 137 (62%) of all persons who died had emigrated from Mexico. Ten (77%) of the black and all 5 of the Asian persons who died were foreign born. At least 1 cysticercosis death was reported from 20 states; California accounted for 57% (126 deaths), and Los Angeles County, California, recorded 32% (70 deaths) of the US total (Figure). Cysticercosis was listed as the primary cause of death for 165 (74.7%) persons.

**Table 1:** Demographic characteristics of 221 patients with fatal cysticercosis, United States, 1990–2002

	No.	%
Sex		
Male	137	62.0
Female	84	38.0
Race/ethnicity		
White	15	6.8
Black	13	5.9
Latino	187	84.6
Asian/Pacific Islander	5	2.3
Native American	1	0.5
Age group, y		
1–4	1	0.5
5–14	5	2.3
15–24	37	16.7
25–34	66	29.9
35–44	29	13.1
45–54	36	16.3
55–64	20	9.1
65–74	15	6.8
75–84	9	4.1
≥85	3	1.4
Education, y*		
<12	135	61.1
12	43	19.5
>12	25	11.3
Country of birth†		
United States	33	14.9
Mexico	137	62.0
Other	50	22.6

*Unknown for 18 persons. †Unknown for 1 person.

**Figure:**
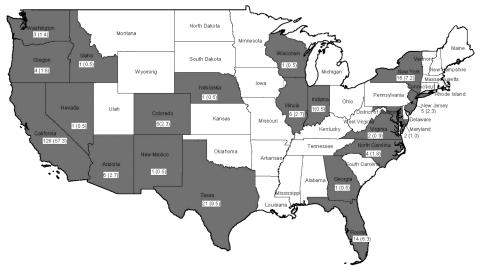
Frequency and percentage of fatal cysticercosis cases by state, United States, 1990–2002. Shaded areas indicate states with deaths from cysticercosis.

Age-adjusted cysticercosis mortality rates were highest for Latinos (adjusted rate ratio [ARR] = 94.5, 95% CI 56.9–56.9, relative to whites) and men (ARR = 1.8, 95% CI 1.4–2.3) (Table 2). The mean age at death was 40.5 years; >60% of deaths occurred in persons <45 years of age. Most persons (61%) had <12 years of education. Although no clear temporal trend was noted, cysticercosis deaths varied by year; most deaths (23) occurred in 1992 and 1997.

**Table 2:** Cysticercosis mortality rates, United States, 1990–2002*

	Rate/10^6^ population (95% CI)	RR (95% CI)
Sex		
Male	0.08 (0.07–0.1)	1.8 (1.4–2.3)
Female	0.05 (0.04–0.06)	Referent
Race/ethnicity		
White	0.006 (0.003–0.008)	Referent
Black	0.03 (0.01–0.05)	5.1 (3.1–8.6)
Latino	0.56 (0.47–0.65)	94.5 (56.9–156.9)
Asian/Pacific Islander	0.04 (0.0–0.07)	6.7 (4.0–11.2)
Native American	0.04 (0.0–0.12)	6.2 (3.7–10.3)

*CI, confidence interval; RR, age-adjusted rate ratio.

The 33 cysticercosis deaths in US-born persons represented 15% of all cysticercosis deaths. Ten (30.3%) of these 33 persons were California residents. US-born persons with fatal cysticercosis had higher educational levels (p = 0.02) and were older (mean age 50.1 vs. 38.7 years, p<0.01) than foreign-born persons with fatal cysticercosis (Table 3). Although 52% of US-born persons who died were Latino, this proportion was lower than that for foreign-born persons (90.4%, p<0.01). At least 1 death of a US-born person was reported in each year of the study period.

**Table 3:** Selected characteristics of US-born and foreign-born persons with fatal cysticercosis, United States, 1990–2002

Characteristic	US-born n = 33 no. (%)	Foreign-born n = 187 no. (%)	p value
Sex			
Male	22 (66.7)	114 (61.0)	0.53
Female	11 (33.3)	73 (39.0)	
Race/ethnicity			
White	12 (36.4)	3 (1.6)	<0.001
Latino	17 (51.5)	169 (90.4)	
Black	3 (9.1)	10 (5.4)	
Asian/Pacific Islander	0 (0)	5 (2.7)	
Native American	1 (3.0)	0 (0)	
Mean age, range	50.1, 2–88	38.7, 7–86	<0.01
Education, y			
<12	12 (36.4)	123 (65.8)	<0.001
12	10 (30.3)	33 (17.7)	
>12	8 (24.2)	17 (9.1)	
Unknown	3 (9.1)	14 (7.5)	

Principal concurrent conditions listed as contributing to death included hydrocephalus in 58 (26.2%) persons, cerebral edema in 23 (10.4%), cerebral compression in 16 (7.2%), and epilepsy/convulsions in 12 (5.4%). These conditions were significantly more common in persons who died of cysticercosis than in matched controls (p<0.001). Septicemia was recorded for 15 (6.8%) of persons with fatal cysticercosis, but this figure was not significant. Reported place of death included inpatient facility (64.7%), emergency room or outpatient clinic (9.5%), nursing home (9.5%), and residence (11.3%).

## Discussion

Our findings indicate that in the United States, cysticercosis is a cause of premature death, particularly among young Latinos, and may be a more frequent cause of death than previously recognized. Substantially more deaths occurred in California, particularly Los Angeles County, and in southwestern states bordering Mexico. Although cysticercosis causes death mainly among Hispanic immigrants, our findings indicate that this larval tapeworm causes infection and death in US-born persons as well.

The elevated cysticercosis mortality rates for Latinos reflect the substantial immigration from *T. solium–*endemic areas of Mexico and other Latin American countries. Over 70% of cysticercosis deaths were of persons born in Mexico. Legal immigration to the United States from Mexico during 1991–2000 was >2.2 million; >1 million additional immigrants came from Central and South American countries (*14*). Moreover, undocumented immigration from such areas continues to occur in considerable numbers. The US Immigration and Naturalization Service estimates that 7 million unauthorized immigrants (4.8 million of these from Mexico) were residing in the United States in January 2000 and that an average of 350,000 immigrate each year (*14*). Cysticercosis and taeniasis are widely prevalent in many Latin American countries. Autopsy studies conducted in Mexico have reported cysticercosis prevalence from 2.8% to 3.6%, and serosurveys have demonstrated infection rates of ≥20% in some areas of Peru, Guatemala, and Bolivia (*3*,*15*). A recent study of farm workers in southern California documented seroprevalence of 1.8% for cysticercosis and 1.1% for taeniasis, comparable to that in cysticercosis-endemic areas (*16*).

We noted several cysticercosis deaths of persons who were born in the United States, which indicates the possibility of locally acquired disease. Transmission of cysticercosis in the United States has been repeatedly documented over the past 20 years and can often be traced to the presence of a tapeworm carrier among household members or other close personal contacts (*3*,*9*–*11*,*17*). An outbreak of neurocysticercosis in an Orthodox Jewish community in New York City implicated domestic employees from Latin America who harbored *Taenia* infections as the probable source of infection (*9*). A pilot surveillance system implemented in Los Angeles County during 1988–1990 identified 10 locally acquired cases among 138 cases reported and found a tapeworm carrier among household contacts for 5 (7%) of 72 overall cases investigated (*10*).

Alternatively, the occurrence of cysticercosis among US-born persons may reflect travel-related exposure and infection. Travel-associated cysticercosis, mainly in persons who have visited Mexico and other Latin American countries, has been previously documented; however the risk and frequency of such infections are unknown (*10*,*18*). The Los Angeles County surveillance system identified 9 probable travel-related cases, which represented 6.5% of the total cysticercosis cases. In a study of cysticercosis in Texas, de La Garza and colleagues reported 6 cases in US-born persons, all of whom had a history of frequent travel to rural Mexico or Central America (*19*). Substantial numbers of US residents travel to cysticercosis-endemic areas each year and may be exposed to food and water contaminated with *T. solium* eggs. Therefore, many of the US-born persons likely acquired infection during travel to endemic areas. Food and water precautions for travelers to cysticercosis-endemic regions should be reinforced.

Although 21 states had at least 1 death from cysticercosis, mortality rates were highest in California and other border states. Cysticercosis deaths were also routinely recorded in New York and Florida. This observed geographic focus of cysticercosis deaths reflects immigration patterns in states that include substantial populations of immigrants from cysticercosis-endemic areas, particularly Mexico and other areas of Latin America.

The sex disparity noted in this study is consistent with data from our recent population study, which demonstrated a significantly higher prevalence of cysticercosis in men (*16*) and likely reflects the greater immigration of young men in search of employment. Such immigration patterns may also explain the relatively young age observed; >60% of cysticercosis deaths in our study were in persons <45 years of age, a heavy toll among young, highly productive persons.

Although we could not assess whether problems with access to healthcare contributed to cysticercosis deaths, >20% of deaths occurred at home, in an emergency room, or in an outpatient setting. Reduced access may have an effect on cysticercosis deaths; additional data on this issue would be useful.

Several large facility-based case series studies have reported that the number of deaths from cysticercosis is relatively low and that the case-fatality rate is <1%. However, such facility-based studies, although providing valuable information, have substantial limitations and may underestimate cysticercosis as a cause of death. Limited data from the pilot Los Angles County surveillance system found a mortality rate of ≈6% (8 of 138 incident cases), and the Dixon study of British troops who had served in India reported mortality rates of nearly 10% (*10*,*20*). However, these case-fatality rates must be viewed with caution because they may reflect underdiagnosis or underreporting of less severe cases and therefore probably represent overestimates. Mortality rates have been reported to be higher for surgically treated patients and those with hydrocephalus, primarily because of increased intracranial pressure and shunt-related infection (*21*). We found that hydrocephalus, cerebral compression/edema, and epilepsy/convulsions were common concurrent conditions recorded on the death certificate. Fatal cysticercosis may also occur in persons who have ingested large numbers of eggs, which may cause an overwhelming, fatal acute infection with numerous larvae and severe central nervous system pathologic changes. Racemose cysticercosis, a phenomenon in which cysticerci continue to grow and proliferate through tissue, may also have a poor prognosis. Newer, less invasive, endoscopic surgical techniques for removing intraventricular cysticerci offer promise of reducing mortality rates (*22*).

Our data, although population based, likely underestimate cysticercosis deaths for several reasons. To be listed on the death certificate, cysticercosis must be recognized and diagnosed, which requires confirmation of infection through biopsy, autopsy, or specialized serologic testing (*23*). Consequently, some cases of fatal cysticercosis likely go undiagnosed and unrecognized, which would result in the miscoding of cysticercosis-related deaths as other conditions. For this reason, death records may be biased and likely underestimate deaths from cysticercosis. The absence of fatal cases reported from Kansas, despite a recent report documenting widespread cysticercosis (*24*), appears to support the notion of underrecognition of fatal cases and suggests caution in interpreting geographic distribution. Our findings demonstrate the benefits of using multiple-cause-of-death data instead of the traditional underlying-cause-of-death data alone for estimating deaths from cysticercosis. An additional 56 (25.3%) cases were identified by using multiple-cause–coded files.

The use of death certificates to assess the effect of disease has advantages and limitations. Because submission of death certificates is required by state law, ascertainment and registration of deaths are virtually complete. Use of mortality records therefore provides population-based data and avoids the potential biases of facility-based data or other data that are not population based. Mortality data can also indicate disease severity and contribute to measures of disease load. However, data from death certificates have several limitations, including the possible coding of inaccurate information through careless completion of cause of death, coding errors, and misclassification of variables such as race/ethnicity (*25*,*26*). Reporting of country of birth may also be inaccurate, and persons with cysticercosis who are recorded as having been born in the United States may, in fact, be foreign born. Deaths from cysticercosis represent only a small fraction of total disease burden. In addition, census data and intercensus population estimates used for the calculation of rates may be uncertain. For these reasons, our estimate of cysticercosis mortality rate must be interpreted with caution.

Cysticercosis can cause severe neurologic disease and death and result in substantial cost to the healthcare system, yet simple public health measures can reduce or eliminate this parasitic disease. In fact, cysticercosis has been identified as 1 of 6 potentially eradicable diseases (*27*). Because most cysticercosis cases in the United States are imported, efforts to control the disease in cysticercosis-endemic regions will reduce disease in the United States. Such control activities can also reduce the likelihood of travel-related infection. State and local health authorities in affected areas of the United States should consider implementing surveillance and follow-up of cysticercosis patients, including attempts to identify and treat tapeworm carriers among household members and other close personal contacts. The availability of a sensitive and specific test for *T. solium* infection that can be performed from blood samples obtained through simple finger stick will facilitate such follow-up (*28*). Given the importance of cysticercosis in border areas, collaborative studies with Mexican public health authorities on the prevalence and incidence of cysticercosis in the border regions should be implemented (*29*,*30*).
